# Comparing Machine Learning Using UAVs to Ground Survey Methods to Quantify Milkweed Stem Density and Habitat Characteristics in ROWs

**DOI:** 10.3390/insects17040359

**Published:** 2026-03-25

**Authors:** Adam M. Baker, Greg Emerick, Christie Bahlai, Scott Eikenbary

**Affiliations:** 1 Davey Institute (Davey Tree Expert Company), Kent, OH 44240, USA; 2 POLLi, Fargo, ND 58102, USA; greg.emerick@polligps.com; 3 Environmental Science and Design Research Institute, Kent State University, Kent, OH 44242, USA; cbahlai@kent.edu; 4 Davey Resource Group (Davey Tree Expert Company), Mount Airy, MD 21771, USA; scott.eikenbary@davey.com

**Keywords:** drone, monarch butterfly, AI, population ecology, vegetation monitoring, field methods, remote sensing

## Abstract

Monarch butterflies have declined in both eastern and western populations. Conservation initiatives that support this imperiled species are being implemented in lands managed by the energy and transportation sectors. Vegetation management strategies that encourage the presence of milkweed (*Asclepias* spp.), the larval host of monarch butterflies (*Danaus plexippus*), or floral resources to support pollinators are being practiced across North America; however, survey methods to evaluate the success of these strategies vary in accuracy and scalability. In this study, we compared five methods to quantify milkweed stem density and land cover estimates: (1) Site al, (2) Transect plot, (3) Square plot, (4) Large transect (informed by the Monarch CCAA methodology), and (5) Machine learning of images collected by UAVs. These methods encompass full coverage ground counts, partial ground counts, and aerial imagery using object-based image analysis. Sites included distribution, transmission, and gas line ROWs, solar arrays, and transportation easements. We found that Site al and Machine learning most consistently quantified milkweed stem density across sites. Partial ground count methods were likely to over or underestimate milkweed populations. Habitat characteristics (woody, broadleaf, grass, and bare ground) estimates were inconsistent across method and site. The intent of this study was to provide land managers with insight as to the most accurate, efficient, and economical approach to quantify milkweed populations and habitat characteristics.

## Simple Summary

Monarch butterfly populations have declined in recent years, increasing the need for conservation efforts led by the U.S. Fish & Wildlife Service, nonprofits, and educational institutions. A key focus of this work is identifying and expanding suitable monarch butterfly habitat, particularly across large landholdings. Utility right-of-way corridors, including electric and gas, solar, and Department of Transportation lands, represent millions of acres of potential habitat for monarchs across North America. This research evaluates five data collection methods that can be used by land managers to identify milkweed, which monarch butterflies need to complete their lifecycle, and habitat features (vegetative and land cover) that support monarchs and other pollinators. Field surveys and drone-based surveys each have clear strengths and limitations. These findings are designed to help land managers determine which approach best fits their operational needs and conservation program goals.

## 1. Introduction

Plant population monitoring is integral to evaluate the effectiveness of integrated vegetation management (IVM) programs [1]. Early detection of risks in rights-of-way (ROWs), such as woody or invasive species encroachment, can provide guidance for management towards compliance, safety, and reliability. However, such programs can also be catered to capture population data on plant communities utilized by beneficial and imperiled species [2,3,4]. Energy corridors are inherently managed in a suspended state of early succession to ensure vegetation does not interfere with energy production or transport. Thus, through active management and intentional cultivation of desirable plants, these habitats have the potential to support invertebrates with high host fidelity and forbs that express unique pollinator syndromes [5,6,7]. One such organism is the monarch butterfly (*Danaus plexippus*), which has declined drastically in both the western [8] and eastern migratory populations [9]. Milkweed (*Asclepias* spp.), the sole larval host for monarchs, thrives in disturbance-driven early successional habitats such as ROWs [10]. In addition, planting milkweed aligns with the IVM strategy of establishing compatible tree-resistant plant cover to reduce invasion of undesirable vegetation [11]. With ~386,000 km of high-voltage lines and millions of km in low-voltage ROWs in the USA [12], and many more available acres in solar, gas, and transportation sectors, there is tremendous potential for such habitats to be managed with an emphasis on conservation [13]. However, plant population assessments of large-scale habitats can be difficult and expensive.

One program that promotes energy and transportation corridors as conservation habitat is the Monarch Candidate Conservation Agreement with Assurances (Monarch CCAA) [14]. Monitoring requires annual inspections of randomly placed 139.4 m^2^ transects (1 per 100 acres) within the adopted lands, where the Pollinator Scorecard is used [15]. The highest tier scorecard records data on milkweed stem density, potential bloom density, native/non-native nectar species, habitat resources, and invasive species. However, milkweeds are not evenly distributed across habitats, making it difficult to capture accurate population data and may lead to over- or under-reporting. Species like common milkweed (*Asclepias syriaca*) grow in clusters due to their reproductive strategy of producing vegetatively via rhizomes [10]. In an aim to address inherent limitations of this methodology, the CCAA began accepting remote sensing data in the summer of 2025. Although the potential of this novel monitoring method is promising, there is little information available for the adoption of this technology in conservation spaces.

Monitoring activities in North American energy corridors are highly decentralized and conducted by diverse groups (government agencies, contractors, university researchers, citizen scientists, non-profits, and internal field technicians), leading to inconsistent methodology and results [16]. There is no consensus or standardization of accepted methods across conservation groups [16,17,18]. Monitoring programs are often limited by spatiotemporal design and logistical/financial considerations, rather than optimal outcomes [19]. In addition, ground surveys can be arduous and often rely on population subsampling and extrapolation [20,21]. Some methods are more appropriate for conspicuous species than inconspicuous ones and others rely on presence/absence data [17]. These challenges make the adoption of machine learning, computer vision techniques, and object-based image analysis of data collected by unmanned aerial vehicles (UAVs) quite attractive for land managers.

UAVs equipped with sensors (RGB, multi-spectral, hyperspectral, thermal) have been used to assess vegetation in agricultural settings [22,23]. This technology has addressed unique agricultural challenges such as weed identification [24], floral structures [25], herbicide injury [26], and abiotic stressors [27,28]. Cases where machine learning successfully identified vegetation characteristics have generally occurred in low-diversity systems that are optimized for crop production and yield. In contrast, ROW habitats are inherently more diverse in botanical composition and habitat characteristics [29]. The complexity of these habitats and the unique metrics required to measure specific conservation or management success indicators are difficult for both ground and UAV surveys to accurately assess at scale. Although there are examples of aerial imagery used in a conservation context [30], it has not been widely adopted in energy and transportation sectors.

Our aim is to evaluate tradeoffs in vegetation monitoring strategies to provide guidance for assessing diverse monarch butterfly habitats in lands managed by the energy and transportation sectors. In this study, we compared various ground survey methods to machine learning algorithms to quantify habitat quality metrics (milkweed stem density and habitat characteristics). Herein, we identify strengths and limitations of each method with the intent of providing land managers with insight into the most efficient, accurate, and economical monitoring approach.

## 2. Materials and Methods

### 2.1. Habitat Characteristics

Milkweed stem density: Ground surveys and machine learning algorithms (analysis of images captured by UAVs) quantified common milkweed stem density within complete or subsample units.

**Figure 1:**
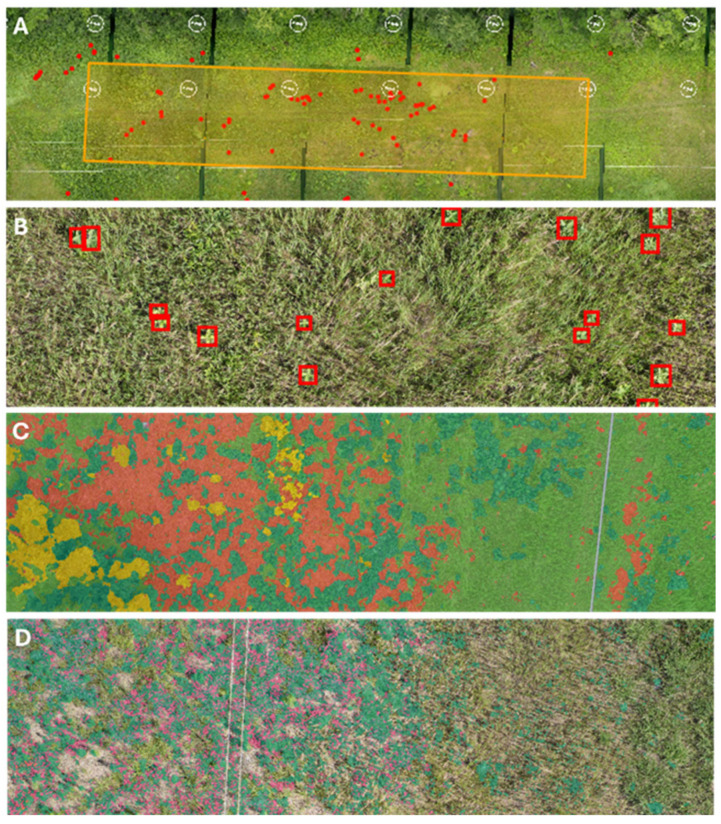
Analysis of habitat characteristics within machine learning software. (**A**) Example of isolated replicate (orange box) in the transmission ROW with milkweed identified (red dots), (**B**) milkweed identification model (red boxes indicating the presence of milkweed stems), (**C**) habitat index model depicting mosaic dense map with habitat features (woody—yellow, grasses—light green, broadleaf—dark green, bare—orange), (**D**) habitat index model depicting floral resources in bloom (magenta) and associated broadleaf plants (dark green).

Habitat characteristics: Ground or algorithmic estimations of canopy cover for complete or subsample units (Figure 1). Categories include (1) floral (plants in bloom), (2) woody (trees/shrubs), (3) grasses, (4) broadleaf (forbs/vines), (5) bare (bare soil/dead vegetation), (6) wetlands, and (7) unclassified (e.g., concrete and gravel).

### 2.2. Ground Truthing

Initial comparisons to assess milkweed stem density were conducted in June 2024 in Chisago County, MN. The Site al and Machine learning methods were performed to quantify milkweed density within the study boundaries. Habitat characteristic data were not collected or analyzed. Approximate time in field was recorded.

### 2.3. Ground Survey Methodology

Site al: The aim of this method is to quantify all milkweed stems within the study boundaries. The observer performed 1 m looping transects throughout the entire study area and recorded all common milkweed stems. All stems that intersected with the observer were counted using clickers. Looping transects were spaced to avoid overlap or double counting. All assessments were performed by the same observer.

Transect plot: The starting position for the transect within each plot was chosen by randomly selecting one of five positions along the narrow edge of the plot. For study sites 2–5, surveyors assessed 2 × 2 m plots at 30.5 m and 61 m along the transect. For study site 6, one plot was assessed at 22.9 m along the transect. For sites 2–5, two plots per replicate were rated (20 per site), and for site 6, one plot was rated (10 per site). Ratings included milkweed stem density and habitat characteristics. Time of field activity was recorded at the completion of each replicate.

Square plot: The starting position for the transect within each plot was chosen by randomly selecting one of five positions along the narrow edge of the plot. For study sites 2–5, surveyors assessed 1 m^2^ subsamples every 10 m (10 per plot/100 total). Five subsamples were assessed at site 6 (Gas ROW), due to limited available area, for a total of 50 subsamples. Subsamples were randomly selected by tossing a 1 m^2^ hoop, alternating from left to right, along the transect. Ratings included milkweed stem density and habitat characteristics. Time of field activity was recorded at the completion of each replicate.

Large transect (informed by Monarch CCAA methodology): As outlined by the Rights-of-way as Habitat working group for Monarch CCAA, which requires annual inspections of one randomly placed 139.4 m^2^ transect per 100 acres, we assessed one transect per study site. We quantified milkweed stem density and habitat characteristics. The transects were randomly assigned in one of the ten plots. Time of field activity was recorded at the completion of each transect. This method is referred to as CCAA in all figures.

### 2.4. Machine Learning Vegetation Assessment

#### 2.4.1. Flight Plans and Data Collection

Before each flight, flags were placed throughout each study area to identify individual replicate boundaries. Flags provided a visual reference within aerial imagery and supported the alignment between aerial observations and ground-based surveys. The replicates were defined in the flight control software (Skydio Autonomy Enterprise version 37.1.166, San Mateo, CA, USA (OH sites) and Auterion Enterprise Suite version 3.7, Arlington, VA, USA Version (MN sites)) before each flight and optimized to minimize unnecessary data collection outside the focal area. Flight paths were planned at the field edge before takeoff to ensure consistent spatial coverage across all study sites.

Flight control systems used standard global positioning systems (GPS) to support navigation, flight stability, sensor triggering, and image metadata recording. A zero-overlap collection strategy captured still images at predefined intervals. This approach ensures 100% observation coverage while reducing the need for excessive data, orthomosaics, stitched imagery, surface reconstructions, or post-flight processing. All images were processed as individual files. No image stitching, mosaicking, or three-dimensional surface modeling was performed. The analysis software identifies overlapped image boundaries to prevent double counting of habitat features.

#### 2.4.2. UAV Platforms and Sensors

Two drones carrying RGB cameras were used for aerial data collection, (1) Freefly Astro UAV (Freefly Systems, Seattle, WA, USA) that held a Sony ILX-LR1 (Sony, Tokyo, Japan) camera equipped with a 50 mm lens was used for all MN sites, and (2) Skydio X10 UAV (Skydio Inc., San Mateo, CA, USA), using their payload of the VT300-L narrow imaging sensor featuring a 64-megapixel sensor and a 50° field of view for all OH sites. The planned flights ensured collision avoidance, maintained proper corridor width, and stayed within designated survey boundaries. The Sony ILX-LR1 platform flew at 33.53 m above ground level (AGL), and the VT300-L platform operated at 30.48 m AGL. Both systems captured data at a ground sampling distance (GSD) of 0.25 cm per pixel, with all missions operated at approximately 5.0 m per second. Image acquisition was configured such that image spacing corresponded to a single image footprint height, resulting in discrete observation points rather than continuous image coverage. The resulting sampling distance between observation points was 15.84 m for the Sony ILX-LR1 system and 17.36 m for the VT300-L system.

#### 2.4.3. Image Analysis

Pre-processing: The drones automatically stamp image metadata into each image. During the upload to the cloud, the software checks the required metadata, which verifies camera position and image quality before an upload can commence. We manually prechecked the image location and quality before uploading the imagery.

Analysis: All UAV imagery was analyzed using POLLi (Fargo, ND, USA), a software platform designed for standardized analysis of high-resolution aerial imagery for vegetation assessment. This tool was used to ingest individual geotagged image frames, perform machine-learning-based inference, and aggregate outputs at the plot and site levels. One image set from each location, all captured at the same 0.25 cm GSD, was used and evaluated using separate inference models. 

#### 2.4.4. Machine Learning Models

Milkweed identification model: This model was used to detect and quantify milkweed stems within individual replicates. This object detection model is designed specifically for common milkweed and was trained with data from multiple sensor types, across multiple geographies, times of day, sun angles, and weather conditions. Other milkweed species that may be present in the ROWs will not be quantified or detected using this model. In the event of data drift, the model relies on a golden dataset (a high-quality dataset that has been ground-truthed for training and evaluating models) for each model to broaden its efficacy across sensors, locations, and developmental stages of the subject plants.

Habitat index model: This model was used to classify habitat features into functional categories in surveyed areas. The semantic segmentation model used to evaluate habitat coverage analyzes an image by assigning a class label to each pixel, producing a dense map that delineates where different objects or land-cover types occur across the scene. This model identifies categories including woody (trees/shrubs), grasses, broadleaf plants (forbs/vines), bare ground/dry vegetation, wetlands, bloom coverage (floral resources), and unclassified (e.g., artificial surfaces). Extensive training across the categories has been conducted using conventional image segmentation techniques at different scales to optimize accuracy and reduce misclassification. A golden dataset was used to manage data drift and maintain the model’s performance at expected levels.

The inference models processed each image separately. The same images used for the milkweed identification model were used for the habitat index analysis. In each image, vegetation detections and classifications were logged along with spatial metadata. The software generated results based on observations calculated by the models inside each digital boundary.

### 2.5. Comparison of Survey Methodologies

**Figure 2:**
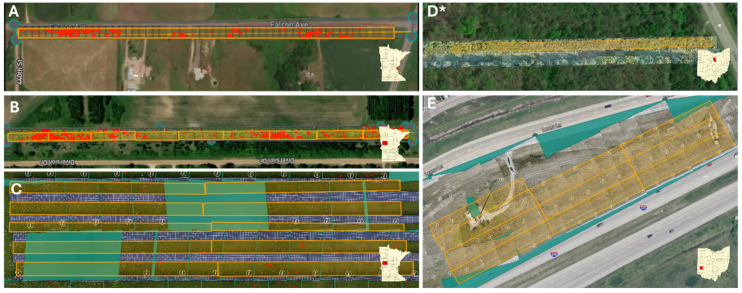
UAV imagery of energy and transportation sites. (**A**) Site 1: Distribution ROW in Chisago Co. MN, (**B**) Site 2: Transmission ROW in Otter Tail Co. MN, (**C**) Site 3: Solar array in Otter Tail Co. MN, (**D**) Site 4: Distribution ROW in Richland Co. OH, (**E**) Site 5: DOT easement in Mongomery Co. OH. * Site 6: Gas ROW adjacent to site 4 in Richland Co. OH.

After machine learning flights were performed, ground surveys were conducted in each plot at each site (Figure 2). This sequencing ensured that vegetation remained undisturbed before the aerial acquisition while maintaining spatial correspondence between aerial imagery and field observations. Data were collected in June in MN and September in OH. Models were trained on all physiological stages of milkweed (seedlings, vegetative early growth, mature stems, stems in bloom, stems with seed pods present, and stems in senescence) to capture temporal variation throughout a given season. Although sampling at different times of the season may influence the detectability of milkweed due to competing vegetative growth, there is no accepted or standardized date for performing milkweed population assessments. Methods were compared relative to the site.

Site 1 (Ground-truthing): We surveyed a 762 m long section of a distribution power line ROW in Chisago County, MN (45°34′56.12″ N, 93°0′6.27″ W). The botanical community consisted of common milkweed, smartweed, mullein, cattails, thistle, sedges, and grasses. Surveys were performed from the outer edge of the power line to the edge of the road (18.3 × 762 m), approximately 2.3 acres. The area consisted of managed meadows with some standing irrigation water on the south end. Assessments were performed on 7 June 2024. For this preliminary ground-truthing assessment, only Site al. and Machine learning (milkweed identification model) were compared.

Site 2 (Transmission ROW): A section of a transmission line ROW in Otter Tail County, MN (46°18′23.82″ N, 96°1′10.23″ W) was surveyed by ground and aerial teams. The 914.4 m long section was divided into ten plots (17.1 × 91.4 m). The botanical community in the ROW consisted of common milkweed, Virginia creeper, wild grape, asters, staghorn sumac, dogbane, and grasses. Assessments were conducted on 26–27 June 2025. All ground survey methods and machine learning models were performed.

Site 3 (Solar array): On 26 June 2025, surveys of a solar array in Otter Tail County, MN (46°16′50.14″ N, 96°1′43.35″ W) with fixed-position panels were conducted. The area was divided into ten plots (6.1 × 91.4 m). The botanical community consisted of common milkweed, butterfly milkweed, asters, yarrow, clover, Canada thistle, sedges, and grasses. All ground survey methods and machine learning models were performed.

Site 4 (Distribution ROW): We surveyed a 914.4 m section of a distribution line ROW in Richland County, OH (40°47′53.0″ N 82°35′07.0″ W). This section was divided into ten plots (15.24 × 91.4 m). The botanical community consisted of common milkweed, grasses, sedges, chicory, asters, smart weed, goldenrod, black berries, Virginia creeper, boneset, and cherries. Assessments were conducted on 9–11 September 2025. All ground survey methods and machine learning models were performed.

Site 5 (Department of Transportation easement): On 9–11 September 2025, we surveyed a Department of Transportation (DOT) easement in Montgomery County, OH (39°63′40.52″ N 84°20′13.72″ W). The area was divided into ten plots (15.24 × 91.4 m). The botanical community consisted of common milkweed, swamp milkweed, cut leaf teasle, cherries, grasses, and sedges. All ground survey methods and machine learning models were performed.

Site 6 (Gas line ROW): Surveys were conducted on a gas line ROW in Richland County, OH (40°47′35.0″ N 82°33′59.1″ W) on 9–11 September 2025. Due to limited access, we divided a 457.2 m long section into ten plots (6.1 × 45.7 m). The botanical community consisted of common milkweed, black berries, saplings (oak, hickory, cherry, maple), and grasses. All ground survey methods and machine learning models were performed.

### 2.6. Cost Estimates

#### 2.6.1. Labor Costs

We quantified labor costs associated with field-based data collection methods using standardized average hourly wage rates. Labor costs were calculated for two common practitioner roles: environmental technicians (field technician) and drone pilots. For each collection method, the total time required to complete data collection in the field was recorded. The national hourly average wage used for analysis was $28.27 [31] for environmental technicians and $28.50 [32] for drone pilots. Time increments were recorded using standard time notation (hh:mm:ss), which represents time as a fraction of a 24 h day. To convert time to billable labor hours, recorded time values were multiplied by 24. The labor cost for each method was calculated using the following formula:

*Hourly Rate* × (*Recorded Time* × 24) = *Cost* *Example*: $28.27 × (1:42:5 × 24) = $48.50

#### 2.6.2. Equipment Costs

Cost estimates for equipment associated with data collection by field personnel were calculated. Standard equipment used by field personnel include a (1) mobile phone/tablet/computer, (2) mid-sized four-wheel-drive pickup truck (including fuel), (3) mobile hotspot (MiFi), and (4) required personal protective equipment (PPE).

To estimate the hourly equipment cost, the total acquisition and operating cost of the listed equipment was summed and then divided by the standard number of full-time work hours per year (1960 h, assuming 40 h per week over 49 working weeks including holiday and PTO). Using this approach yields an estimated equipment cost of $18.43 per hour, which represents the average hourly cost of providing and maintaining essential field equipment for a single practitioner/field worker.

To illustrate how equipment costs were incorporated into overall labor costs, the example below combines the calculated hourly equipment cost with the national hourly average wage. The combined hourly rate (labor + equipment) was then applied using the same approach described above. Recorded field time values were converted to billable labor hours by multiplying the observed time by a factor of 24, allowing for standardized comparison across data-collection methods.

*Hourly Rate* × (*Recorded Time* × 24) = *Cost* *Example*: $38.50 + 18.43 = $56.93 $56.93 × (1:42:5 × 24) = $97.67

#### 2.6.3. UAV Equipment Costs and Analysis

In addition to the standard field equipment required for a field technician, a drone pilot must be equipped with UAV-specific equipment necessary for aerial data acquisition. This equipment includes a drone, a high-resolution camera and lens, batteries, a battery charging system, and a travel case for safe mobilization. Drone-based data acquisition also requires post-collection analytical tools to convert imagery into usable data. These resources include imagery processing and analysis software, data storage, and annual software licensing fees.

To estimate an hourly cost of UAV-related equipment and analytical resources, the total cost of standard field equipment, UAS hardware, and imagery analysis and data storage was summed and then divided by the standard number of full-time work hours per year (1960 h, assuming 40 h per week over 49 working weeks, accounting for holidays and paid leave). Using this approach yields an estimated combined equipment and analytical cost of $35.02 per hour. This represents the average hourly cost of providing and maintaining the essential field equipment, UAS tools, and data-processing required to support this type of data collection and machine-learning-assisted analysis.

*Drone Pilot Hourly Rate*
+ *Field Equipment* + *UAS Hardware* & *Machine Learning*
× (*Recorded Time* × 24) = *Cost* *Example*: $28.50 + 18.43 + 16.59 = $63.52 $63.52 × (1:42:5 × 24) = $108.0

### 2.7. Statistical Analysis

Differences in milkweed density among survey methods and sites were evaluated using two-way ANOVA models with the method, site, and their interaction as fixed effects. When significant effects were detected, post hoc comparisons were conducted using pairwise *t*-tests within sites. Because the CCAA method was not replicated within sites, comparisons involving CCAA were evaluated using single-sample *t*-tests. Bonferroni adjustments were applied to all post hoc tests to control the family-wise error rate. Milkweed density results are reported as mean estimated stems per hectare. The spatial structure among subsamples reflects the sampling design of each survey method and was treated as part of the methodological differences being evaluated.

To assess similarity among milkweed density estimates produced by different methods, we conducted correlation analyses using plot-level data pooled across sites. Pairwise method comparisons were evaluated using linear regression, and the slope and coefficient of determination (R^2^) were extracted for each comparison. Slopes approaching 1 indicate a similar magnitude of estimates between methods, while R^2^ values near 1 indicate consistent relationships.

To evaluate method performance relative to a reference estimate of milkweed stem density, we treated Site al as the best approximation of true density. For each plot within each site, we calculated the absolute difference between Site al and each other method. We then quantified the proportion of observations falling within the interquartile range of Site al estimates, as well as the proportions that over- or underestimated relative to this reference.

Patterns of habitat characteristics were analyzed separately from milkweed density. Because Site al did not provide groundcover estimates, it was excluded from these analyses. To test whether survey methods produced similar distributions of groundcover types across sites, chi-square goodness-of-fit tests were conducted within sites for the categories floral, woody, grass, broadleaf, and bare. Wetland and unclassified categories were excluded due to low detection or method-specific absence. For each groundcover type, two-way ANOVAs with the method, site, and their interaction were used to assess differences among methods, followed by post hoc pairwise and single-sample *t*-tests with Bonferroni correction as described above.

Consistency among methods in estimating groundcover was further assessed using pairwise linear regressions for each groundcover type, pooling data across sites and plots. For each method pair, slopes and R^2^ values were calculated and summarized in tables to quantify agreement. Finally, agreement among groundcover methods was evaluated relative to Machine learning as a baseline by calculating the proportion of plot-level observations that overestimated, underestimated, or agreed with baseline estimates.

All analyses and visualizations were conducted in R v4.5.2 [33] using packages including tidyverse [34], ggplot2 [35], broom [36], cowplot [37], ggbreak [38], and multcompView [39].

## 3. Results

### 3.1. Ground Truthing

In this initial assessment, only the milkweed stem density was recorded. Ground surveys, employing the Site al method, recorded 579 milkweed stems. Machine learning analysis of UAV-collected images identified 503 milkweed stems (86% of total stems identified in the ground survey) within the study area boundaries. The ground survey took ~120 min, whereas UAV image collection took ~30 min.

### 3.2. Comparison of Survey Methodology to Detect Milkweed Stem Density

**Figure 3:**
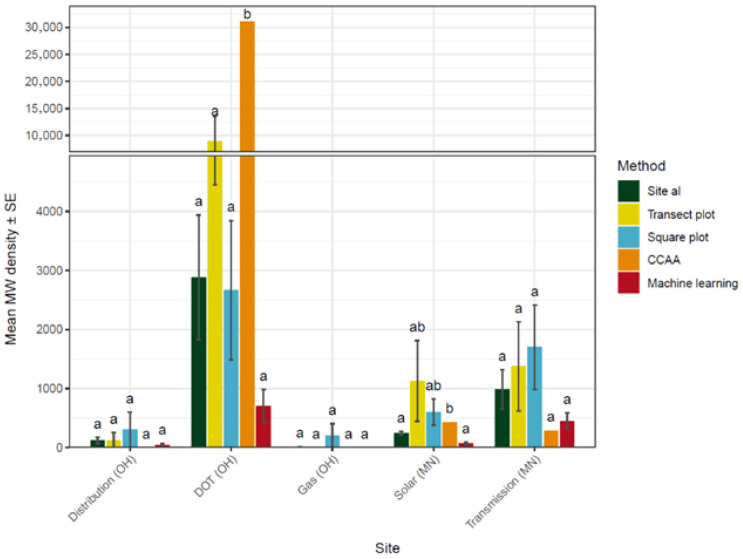
Mean milkweed stem density estimates (±SE) derived from five survey methods across study sites. Estimates are summarized at the site level, with letters indicating significant pairwise differences among methods within sites based on post hoc tests. A broken y-axis is used to facilitate comparison across methods given the large variation in estimated densities.

Estimated milkweed density varied by site (F_4,178_ = 10.9, *p* < 0.001) and by survey method (F_4,178_ = 4.9, *p* < 0.001), but the interaction of site by method was also significant (F_16,178_ = 4.3, *p* < 0.001), indicating that patterns of milkweed density captured by each method varied between sites (Figure 3). Post hoc tests did not detect differences between methods at Distribution, Gas or Transmission, but this pattern is partially driven by extreme variation within some methods at several sites, especially Distribution and Gas.

Overall model fit was poor, likely due to the patchy spatial distribution of milkweed and the resulting non-normal detection patterns across survey methods. The results nonetheless highlight systematic differences in how survey methods estimate milkweed density.

**Figure 4:**
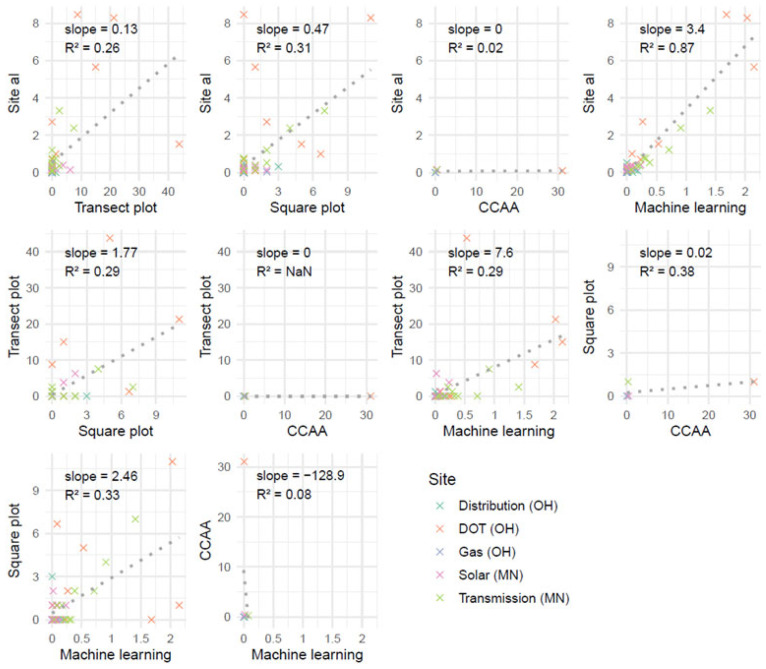
Scatterplot matrix of pairwise comparisons among survey methods for milkweed stem density. Points represent individual plots and are colored by site. Dotted lines show linear regressions, with slope and R^2^ values annotated in each panel. Axes are in units of thousands of stems per hectare.

Pairwise comparisons of milkweed stem density estimates among survey methods suggested that Site al and Machine learning produced the most consistent results across plots (R^2^ = 0.87) (Figure 4). However, Site al consistently recorded approximately three times higher milkweed densities than Machine learning. Among the replicated field methods, Square plot estimates were generally closest to those from Site al and Transect plot (i.e., Square plot estimated milkweed densities at about half the rate of Site al and nearly twice the rate of Transect plots), although the relationships were weak and variable (R^2^ = 0.31 and 0.29, respectively), indicating that additional sampling may be required for reliable density estimates. The CCAA method showed little correspondence with other methods, likely reflecting its reliance on a single transect per site and the resulting lack of replication.

**Figure 5:**
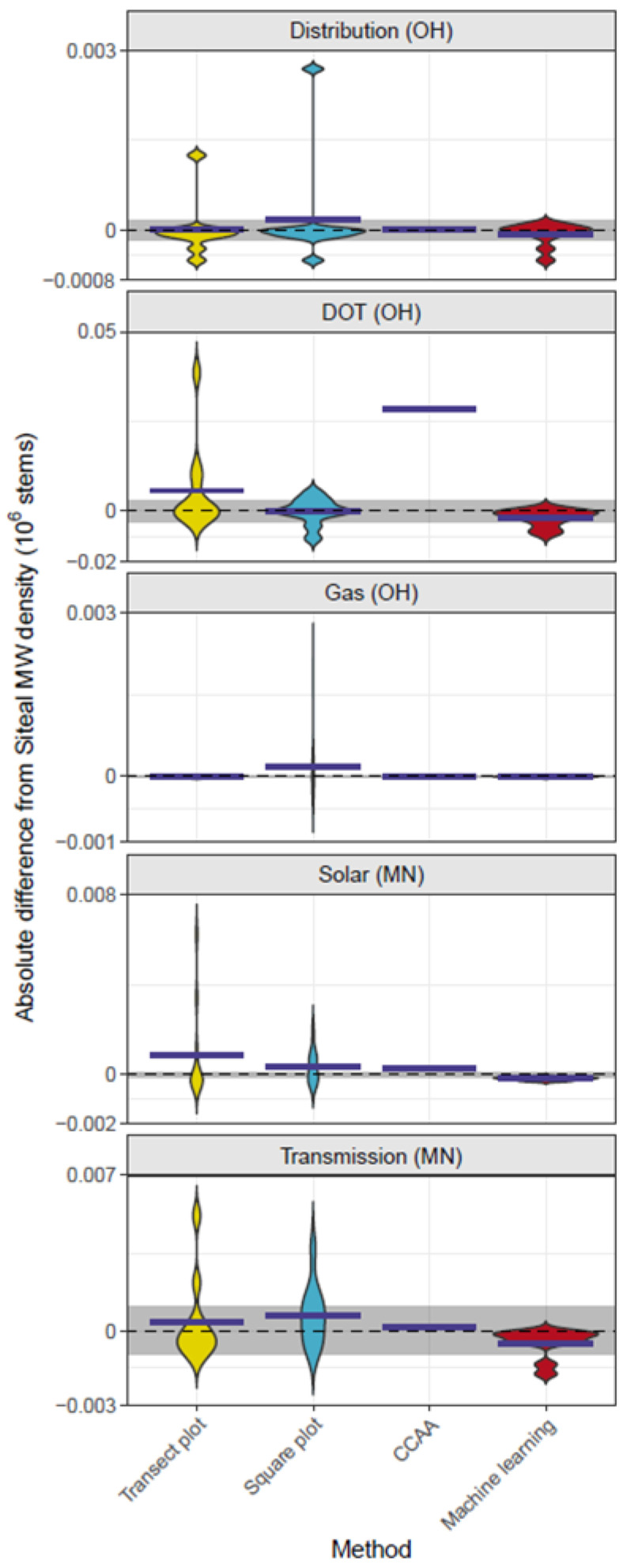
Violin plots showing absolute differences in milkweed density estimates by method relative to Site al across sites. The dashed line at zero indicates the Site al reference, with the gray ribbon showing ±1 SD. Dark blue ticks mark the site-level mean difference for each method.

Differences from Site al milkweed stem density estimates varied systematically across methods (Figure 5). Transect plots and Square plots often overestimated milkweed density, though the distributions of estimates were broad due to occasional high-density patches inflating site-level means. However, individual measurements capturing low densities of milkweed were frequently observed in Transect plots and Square plots, reflecting the influence of rare high-density patches captured in these methods. CCAA estimates were highly variable, falling above, below, or near Site al values. Machine learning consistently underestimated Site al densities, but the deviations were narrowly distributed.

### 3.3. Comparison of Survey Methodologies to Estimate Habitat Characteristics

The distribution of landcover categories estimated by each method differed significantly at all sites. Chi-square tests confirmed that these differences were significant, with X^2^ values ranging from 1225 to 28,340 (12 degrees of freedom, all *p* < 0.001), indicating that methods capture different patterns of ground cover within each site.

**Figure 6:**
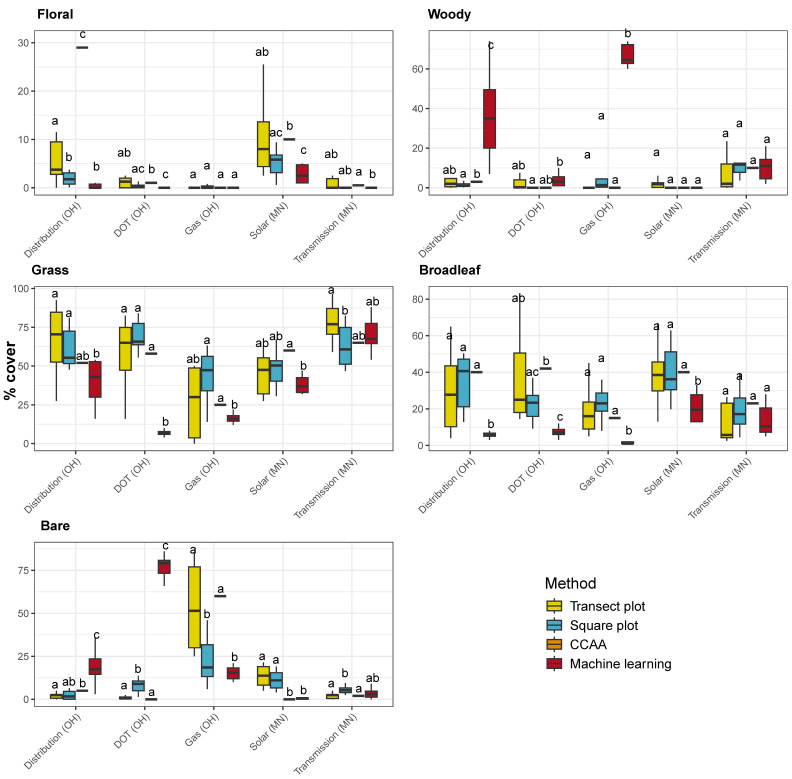
Estimated proportions of each groundcover type captured by different survey methods across sites. Boxes represent the distribution of observations for floral, woody, grass, broadleaf, and bare cover. Letters indicate significant differences between methods within each site based on post hoc pairwise *t*-tests and single-sample *t*-tests with Bonferroni adjustment.

Within each site, survey methods captured ground cover differently (Figure 6). ANOVAs for each groundcover type showed significant variation by method and site, and the relative patterns among methods were not consistent across sites (*p* < 0.001 in all cases). Machine learning was the only method to consistently detect woody cover, suggesting either a broader sampling of woody patches by this method or greater sensitivity to sparse vegetation. Additionally, Machine learning consistently recorded lower estimates of grass and broadleaf cover compared to other methods.

Overall, no two methods produced identical estimates of groundcover (Table 1). Machine learning generally showed patterns most similar to Transect plot and Square plot methods, with the notable exception of woody cover, which it captured uniquely, and bare ground, where relationships with other methods were sometimes negative. In contrast, CCAA exhibited inconsistent relationships with all other methods across groundcover types, suggesting it provides less reliable or less comparable estimates.

Probabilities of falling within the Site al interquartile range show that Machine learning did so roughly half the time, while Transect plots, Square plots, and CCAA could overestimate, underestimate, or fall within the range (Table 2). Across sites, the three in-field survey methods showed broadly similar patterns in their deviations from Machine learning estimates. All three methods were most likely to overestimate floral, grass, and broadleaf groundcover, while tending to underestimate woody and bare groundcover relative to the baseline. Agreement with Machine learning estimates was highest for floral cover, indicating that methods capture this category more consistently than others.

### 3.4. Comparison of Cost and Efficiency of Field Activities

The cost of field activities varied by site and method (Table 3). Of the 100% coverage methods, Site al ($404.65 total) tended to cost more than Machine learning ($311.24 total). Average hourly labor estimates were similar between Site al and Machine learning ($28.27 for field technicians and $28.50 per hour for drone pilots). However, hourly equipment costs ($18.43 for ground surveys and $35.02 for UAV data collection per hour) were higher for drone/machine learning-related activities. Of the subsample ground survey methods (Transect plot, Square plot, and CCAA), Square plot tended to be the most expensive and CCAA the least ($221.54, $350.40, and $49.03 respectively).

Time to complete vegetation assessments in the field varied by site and method (Table 4). Site al generally took the most time to complete (08:30:43 total time) but was faster than subsample methods in low-diversity ROWs (e.g., Gas). Of the subsample methods (Transect plot, Square plot, and CCAA), Square plot took the most time to complete (04:43:54, 07:27:43, and 01:03:00 respectively). The CCAA method took the least amount of time. Machine learning (01:41:20) was similar to CCAA and more efficient compared to other ground survey methods. Automated processing times of the milkweed identification model and habitat index model reflect the time after upload and are not included in field activities.

## 4. Discussion

The vast network of lands managed by the energy and transportation sectors has the potential to support the conservation of plants [40], insects [2,4], birds [41], and mammals [42]. The linear nature of ROWs inherently creates connectivity between diverse habitats, which may improve the ability of breeding monarchs to find suitable habitats. In addition, these linear spaces may be used as travel corridors for migrating species such as monarch butterflies. With numerous conservation certification programs promoting the use of ROWs for supporting imperiled species [43,44], there is an increasing need for monitoring populations of concern at scale. This study provides comparative insight into the accuracy, cost, and efficiency of five methods to quantify milkweed populations and habitat characteristics in ROWs to support decision-making, and further efforts to protect monarch butterflies, which are under consideration for listing as a threatened species by USFWS [45].

The use of machine learning to analyze aerial photos to measure conservation metrics is not yet widespread. Color (RGB) digital imagery, the most common and inexpensive payload for any UAV, provides results on vegetative cover similar to that of more expensive sensors (e.g., hyperspectral and multispectral) [23]. Application of remote sensing tools at scale need to be economically viable and comparably accurate to ground methods for adoption. Standardizing data collection reduces time and cost by enabling the same digital images to be used across multiple inference models. The potential to train machine learning models on new species of conservation concern, invasive species/non-compatible plants, and novel habitat features will continue to increase the value and scope of this technology as a vegetation management and population estimate tool.

In this study, Machine learning underestimated milkweed stem density but in a very consistent way compared to more labor-intensive, whole or partial counting methods. Site al consistently found approximately three times more milkweed stems compared to Machine learning, suggesting this method is most able to capture cryptic, clustered or small milkweed stems, which may not be detected when habitats are complex. Counts from full ground surveys may help inform population estimates of the models, particularly for complex habitats. In our initial ground truthing, which was performed in the early season, milkweed stems were generally taller than competing vegetation. In this simplified habitat, the milkweed identification model was 86% accurate compared to Site al. Early season assessments may be advantageous for capturing vegetative basal regrowth (and provide high levels of accuracy) but may miss milkweed stems sprouting from seed that will occupy canopy space later in the season. In our scaled-up experiment, habitats were more complex and measurements were taken over the growing season, and despite the lower detection rates, Machine learning was strongly correlated with Site al estimation.

All of the methods to evaluate land cover characteristics performed differently at each site. Of the habitat characteristics examined, floral coverage estimates were most consistent across all methods. Woody cover was captured by Machine learning at higher rates compared to ground methods. This is likely due to the perspective of the images, which capture overhanging canopy or taller stems that did not intersect with subsample ground transects.

We also evaluated trade-offs of cost and information quality across all the methods. While Machine learning was middling in overall cost compared to the other methods, this method provides distinct advantages in the field. Ground survey teams face their own suite of challenges. ROWs with diverse habitat characteristics can be difficult to safely traverse. Each field visit presents a risk to personnel who may encounter dangerous situations including uneven terrain, arthropods (wasps, mosquitoes, and ticks), heat-related stress, vehicular incidents, and overhead hazards [46,47]. Multiple site visits or repetitively performing the same task may lead to complacency [48] and result in over- or under-counting. Fatigue due to heat and repetition may also influence the accuracy of field surveys [49].

**Figure 7:**
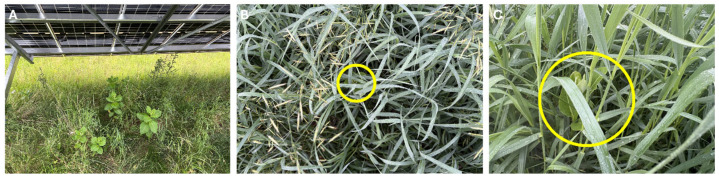
Obstructed milkweeds in ROWs. (**A**) Milkweed stems underneath solar panels that were not captured in aerial imagery due to obstruction, (**B**) milkweed stem obscured by grass from a bird’s eye perspective (yellow circle), (**C**) obscured milkweed stem from a ground surveyor’s perspective (yellow circle).

Although the machine learning algorithms performed consistently in ROW settings, the UAVs capturing the images have some inherent limitations. Trees, infrastructure, and other obstacles can make acquisition flights impossible or unsafe at some locations. For example, it was difficult in the solar arrays to fully assess vegetation due to blockages by fixed-position panels or the deep shadows they cast (Figure 7). Machine learning was able to identify 38 milkweed stems between the panels, whereas the Site al recorded 135 stems. Furthermore, technical problems with data acquisition may not be detected until after sampling is complete. For instance, during this same acquisition flight, the sensor failed to trigger, resulting in two missing photos. Yet, sampling errors were rare: this was the only flight where the sensor malfunctioned. In addition, 110 milkweed stems were recorded underneath the panels adjacent to the survey boundaries, which were counted separately by ground surveyors.

UAV images are captured from a bird’s eye view; thus, plants of interest that are obstructed by structures or competing vegetation may not be counted. Milkweed seedlings, multi-stemmed or close-growing milkweeds, stems intermixed with other non-target vegetation, stems in later stages of senescence, or mechanically damaged stems may be missed by inference models. Although milkweed stems obscured by competing vegetation may be missed by machine learning models, such stems are less likely to be used by monarchs for oviposition. Studies looking at oviposition dynamics by monarchs suggest that isolated plants with strong visual silhouettes [50] or milkweeds on the edge of plantings are preferred [51].

Ground surveyors can detect plants that aerial imagery may overlook due to their multi-angle perspective, possibly explaining the disparity over Machine learning results. The Site al method accounted for the greatest milkweed abundance, which is unsurprising due to its intensive approach, and was consistent in its relative relationship to Machine learning. Of the ground survey methods, Site al was generally the most time-consuming and costly. Thus, this method may be impractical for large tracks of habitat due to efficiency and the risk to field personnel. At the opposite extreme, we found that CCAA, an accepted and practiced approach, took the least amount of time/expense and was the most practical to implement in the field, but was most likely to severely over- or underestimate milkweed or habitat characteristics. For example, at the DOT site, the randomly placed CCAA plot landed on a dense milkweed patch, accounting for 433 stems, resulting in drastic overestimation compared to other methods (Figure 3). Moreover, because of the single-sample approach per site this estimate provides, it is impossible to quantify the uncertainty of estimates. Systematic subsampling of habitats was more efficient and less expensive than full-coverage ground counts and more accurate than CCAA. Between these methods, we found that the Transect plot was more efficient than the Square plot and more similar to other methods than CCAA. However, this method is likely to produce variable results and was likely to over/underestimate in all characteristics compared to Machine learning. The Square plot was similar in its performance to the Transect plot but was much less efficient.

One opportunity that ground surveys offer is the addition of insect counts/collections during field visits, such as the Pollard cube method [52]. Studies evaluating insect identification using machine learning models exist but generally depend on public datasets (e.g., iNaturalist) or fixed position cameras [53]. Acquisition of unique insect image datasets that adequately cover large-scale habitats is difficult, and they are expensive to collect/analyze. High-resolution images of insects on the wing or that are moving between plants in the canopy are difficult to capture.

## 5. Conclusions

All the approaches evaluated in this study have their inherent limitations and strengths. Although we did not explicitly test a hybrid approach, some locations (e.g., solar arrays) can benefit from both UAV and ground assessments. Large areas can be flown cost-effectively, and machine learning can provide adequate estimates, using the same set of images to assess multiple features. Machine learning also provides an interactive image-based reference of all data captured, whereas ground surveys generally result in numerical and categorical data. Areas with critical infrastructure, encroachment by woody species, discrete plant material of interest, or sections of the ROW where enhancements, such as pollinator-centric plants, have been established may be more appropriate for a well-trained ground surveyor.

## Figures and Tables

**Table 1:** Pairwise comparisons of groundcover estimates between survey methods for each habitat characteristic. For each method pair, linear models were fitted to the cover estimates of each characteristic (floral, woody, grass, broadleaf, bare) across all sites and plots. Slopes indicate the relative magnitude of estimates between methods, with values approaching 1 representing similar quantification, and R^2^ values indicate the consistency of the relationship. For fitting regressions, Method 1 was treated as the independent variable and Method 2 was treated as the dependent variable.

		Groundcover Type									
		Floral		Woody		Grass		Broadleaf		Bare/Dead Vegetation	
Method 1	Method 2	Slope	R^2^	Slope	R^2^	Slope	R^2^	Slope	R^2^	Slope	R^2^
Machine learning	Transect plot	2.6	0.41	0	0	0.5	0.18	0.6	0.07	−0.2	0.04
Machine learning	Square plot	1.4	0.74	0.1	0.06	0	0	0.6	0.12	0	0.01
Machine learning	CCAA	19	0.71	0	0	0.4	0.3	1	0.14	−0.1	0.02
Transect plot	Square plot	0.2	0.36	0.9	0.36	0.4	0.42	0.4	0.3	0.3	0.56
Transect plot	CCAA	0.5	0.05	0.4	0.89	0.5	0.69	0.9	0.68	0.7	0.93
Square plot	CCAA	11.2	0.93	0.4	0.95	0.9	0.56	0.4	0.15	1.4	0.81

**Table 2:** Probability of overestimation, underestimation, or agreement with reference to Site al for milkweed stem densities and Machine learning for groundcover type by method. For each survey method, the proportions of plot-level estimates that fall above, below, or within the interquartile range (25th–75th percentile) of Site al densities and Machine learning groundcover estimates are reported. These probabilities quantify method bias and accuracy relative to the reference.

		Probability of Outcome		
Characteristic	Method	Underestimate	Agreement	Overestimate
Milkweed	Transect plot	0.54	0.24	0.22
	Square plot	0.44	0.3	0.26
	CCAA	0.2	0.4	0.4
	Machine learning	0.5	0.5	0
Floral	Transect plot	0	0.5	0.5
	Square plot	0.02	0.46	0.52
	CCAA	0	0.2	0.8
Woody	Transect plot	0.66	0.12	0.22
	Square plot	0.64	0.3	0.06
	CCAA	0.6	0.4	0
Grass	Transect plot	0.16	0.14	0.7
	Square plot	0.2	0.06	0.74
	CCAA	0	0.2	0.8
Broadleaf	Transect plot	0.16	0.06	0.78
	Square plot	0.04	0.12	0.84
	CCAA	0	0	1
Bare	Transect plot	0.46	0.14	0.4
	Square plot	0.46	0.14	0.4
	CCAA	0.6	0.2	0.2

**Table 3:** Cost estimates for habitat assessments in the field. Labor estimates calculated using national averages for field technicians and drone pilots. Standard equipment costs were used for both labor types, whereas UAV-specific costs were used for only the drone pilot. Image analysis based on $0.57 per image. Costs do not include project management, mobilization, report writing, and other aspects of services.

Site	Method	Field Technician ^1^	Drone Pilot ^2^	Equipment ^3^	Image Analysis ^4^	Total Cost
Transmission	Site al	$72.81	------	$47.47	------	$120.28
Transmission	Transect plot	$17.28	------	$11.27	------	$28.55
Transmission	Square plot	$31.40	------	$20.47	------	$51.88
Transmission	CCAA	$4.58	------	$2.98	------	$7.56
Transmission	Machine learning	------	$9.53	$11.71 *	$35.24	$56.48
Solar	Site al	$48.50	------	$31.62	------	$80.12
Solar	Transect plot	$40.71	------	$26.54	------	$67.25
Solar	Square plot	$51.51	------	$33.58	------	$85.10
Solar	CCAA	$6.29	------	$4.10	------	$10.39
Solar	Machine learning	------	$8.07	$9.91 *	$21.09	$39.07
Distribution	Site al	$53.23	------	$34.70	------	$87.94
Distribution	Transect plot	$28.63	------	$18.67	------	$47.30
Distribution	Square plot	$42.20	------	$27.51	------	$69.71
Distribution	CCAA	$3.86	------	$2.51	------	$6.37
Distribution	Machine learning	------	$8.17	$10.04 *	$70.68	$88.89
DOT	Site al	$63.74	------	$41.55	------	$105.30
DOT	Transect plot	$33.85	------	$22.07	------	$55.92
DOT	Square plot	$46.00	------	$29.99	------	$75.99
DOT	CCAA	$12.86	------	$8.39	------	$21.25
DOT	Machine learning	------	$10.73	$13.18 *	$47.31	$71.22
Gas	Site al	$6.67	------	$4.35	------	$11.01
Gas	Transect plot	$13.63	------	$8.89	------	$22.52
Gas	Square plot	$37.97	------	$24.75	------	$62.72
Gas	CCAA	$2.10	------	$1.37	------	$3.46
Gas	Machine learning	------	$11.64	$14.30 *	$29.64	$55.58

^1^ Based on average hourly wage for field technicians; ^2^ Based on average hourly wage for drone pilots; ^3^ Standard equipment costs associated with field activities (PPE, vehicle, computer, mobile hotspot); ^4^ Based on image analysis cost of $0.57 per image (0.57 × # of images); * Additional UAV-specific equipment cost for a drone pilot (drone/camera/batteries/subscription fees) combined with standard equipment cost.

**Table 4:** Time associated with habitat assessments in the field. Time in the field and processing times presented as totals. Time per unit (subsamples) presented as means. All data reported in standard time notation (hh:mm:ss).

				Processing Time	
Site	Method	Time in Field	Time per Unit (# of Units)	Milkweed ID Model	Habitat Index Model
Transmission	Site al	02:24:32	00:15:27 (10)	------	------
Transmission	Transect plot	00:40:40	00:02:02 (20)	------	------
Transmission	Square plot	01:06:39	00:00:40 (100)	------	------
Transmission	CCAA	00:09:43	00:09:43 (1)	------	------
Transmission	Machine learning	00:20:04	00:02:01 (10)	00:22:46	00:41:09
Solar	Site al	01:42:56	00:10:17 (10)	------	------
Solar	Transect plot	01:26:24	00:04:19 (20)	------	------
Solar	Square plot	01:48:47	00:01:06 (100)	------	------
Solar	CCAA	00:13:21	00:13:21 (1)	------	------
Solar	Machine learning	00:16:59	00:01:41 (10)	00:23:51	00:41:11
Distribution	Site al	01:52:59	00:11:17 (10)	------	------
Distribution	Transect plot	00:56:03	00:03:02 (20)	------	------
Distribution	Square plot	01:41:00	00:01:00 (100)	------	------
Distribution	CCAA	00:08:11	00:08:11 (1)	------	------
Distribution	Machine learning	00:17:12	00:01:43 (10)	00:23:51	00:41:11
DOT	Site al	02:14:32	00:13:31 (10)	------	------
DOT	Transect plot	01:11:51	00:03:36 (20)	------	------
DOT	Square plot	01:37:38	00:00:59 (100)	------	------
DOT	CCAA	00:27:18	00:27:18 (1)	------	------
DOT	Machine learning	00:22:35	00:02:16 (10)	00:26:13	00:42:39
Gas	Site al	00:15:44	00:01:32 (10)	------	------
Gas	Transect plot	00:28:56	00:02:53 (10)	------	------
Gas	Square plot	01:13:30	00:01:37 (50)	------	------
Gas	CCAA	00:04:27	00:04:27 (1)	------	------
Gas	Machine learning	00:24:30	00:02:27 (10)	00:23:11	00:43:21

## Data Availability

The data is available upon reasonable request by the corresponding author.
